# A promising approach for screening pulmonary hypertension based on frontal chest radiographs using deep learning: A retrospective study

**DOI:** 10.1371/journal.pone.0236378

**Published:** 2020-07-24

**Authors:** Xiao-Ling Zou, Yong Ren, Ding-Yun Feng, Xu-Qi He, Yue-Fei Guo, Hai-Ling Yang, Xian Li, Jia Fang, Quan Li, Jun-Jie Ye, Lan-Qing Han, Tian-Tuo Zhang

**Affiliations:** 1 Department of Pulmonary and Critical Care Medicine, The Third Affiliated Hospital of Sun Yat-sen University, Institute of Respiratory Diseases of Sun Yat-sen University, Guangzhou, China; 2 Center for Artificial Intelligence in Medicine, Research Institute of Tsinghua, Pearl River Delta, Guangzhou, China; 3 Department of Medical Ultrasound, The Third Affiliated Hospital of Sun Yat-sen University, Guangzhou, China; 4 Department of Radiology, The Third Affiliated Hospital of Sun Yat-sen University, Guangzhou, China; 5 Department of Pulmonary and Critical Care Medicine, The Third Affiliated Hospital of Sun Yat-sen University, Yuedong Hospital, Meizhou, China; 6 Department of Pumonary Diseases, Dongguan Tangxia Hospital, Dongguan, China; Newcastle University, UNITED KINGDOM

## Abstract

**Background:**

To date, the missed diagnosis rate of pulmonary hypertension (PH) was high, and there has been limited development of a rapid, simple, and effective way to screen the disease. The purpose of this study is to develop a deep learning approach to achieve rapid detection of possible abnormalities in chest radiographs suggesting PH for screening patients suspected of PH.

**Methods:**

We retrospectively collected frontal chest radiographs and the pulmonary artery systolic pressure (PASP) value measured by Doppler transthoracic echocardiography from 762 patients (357 healthy controls and 405 with PH) from three institutes in China from January 2013 to May 2019. The wohle sample comprised 762 images (641 for training, 80 for internal test, and 41 for external test). We firstly performed a 8-fold cross-validation on the 641 images selected for training (561 for pre-training, 80 for validation), then decided to tune learning rate to 0.0008 according to the best score on validation data. Finally, we used all the pre-training and validation data (561+80 = 641) to train our models (Resnet50, Xception, and Inception V3), evaluated them on internal and external test dataset to classify the images as having manifestations of PH or healthy according to the area under the receiver operating characteristic curve (AUC/ROC). After that, the three deep learning models were further used for prediction of PASP using regression algorithm. Moreover, we invited an experienced chest radiologist to classify the images in the test dataset as having PH or not, and compared the prediction accuracy performed by deep learing models with that of manual classification.

**Results:**

The AUC performed by the best model (Inception V3) achieved 0.970 in the internal test, and slightly declined in the external test (0.967) when using deep learning algorithms to classify PH from normal based on chest X-rays. The mean absolute error (MAE) of the best model for prediction of PASP value was smaller in the internal test (7.45) compared to 9.95 in the external test. Manual classification of PH based on chest X-rays showed much lower AUCs compared to that performed by deep learning models both in the internal and external test.

**Conclusions:**

The present study used deep learning algorithms to classify abnormalities suggesting PH in chest radiographs with high accuracy and good generalizability. Once tested prospectively in clinical settings, the technology could provide a non-invasive and easy-to-use method to screen patients suspected of having PH.

## Introduction

Pulmonary hypertension (PH), a pathophysiological disorder that may involve multiple clinical conditions, is defined as an increase in mean pulmonary arterial pressure (PAP) at rest status as assessed by right heart catheterization (RHC). It can complicate a series of cardiovascular and respiratory diseases, and leads to right heart failure and even death if left untreated [1–3]. Though the signs and symptoms of PH are nonspecific, a slightly elevated PAP may have adverse prognostic implications in patients [4–6]. Because of the poor prognosis it causes, it’s of great significance to screen patients with PH at an early stage for timely intervention or to assess the value of PAP for following up patients with cardiac or pulmonary disorders [1]. RHC is considered to be “gold standard” for quantification of PAP as it can directly measure the pressure [7]. However, it is invasive and associated with a small but well-defined risk [8, 9]. Noninvasive assessment by doppler transthoracic echocardiography (TTE) is recommended by the current guidelines as an initial screening test for PH [10]. Noninvasive diagnosis of PH with TTE has good sensitivity, specificity, and accuracy for a pulmonary artery systolic pressure (PASP) cut-off value of 40 mmHg [1,11]. Thus, TTE has been proved to be a reliable method for assessment of PASP, and is well-suited to establish a noninvasive diagnosis of PH. However, owing to a shortage of equipment and a lack of experienced echocardiography experts in poor areas of developing countries, it is difficult to screen asymptomatic patients with TTE in a regular health examination. This difficulty may impair screening efficacy and work-up efforts. Chest X-rays (CXRs) are often considered as the first step in medical examinations of organs in the chest, as it’s quickly and easily obtained besides lower dose of radiation exposure [12]. The presentation of PH in CXRs include enlargement of the central pulmonary arteries, with or without rapid tapering (pruning), and right heart chamber enlargement [13,14]. Nevertheless, CXRs are known to be insensitive for detection of PH, and a normal CXR does not exclude PH. Physicians other than radiologists may have difficulty in making accurate diagnoses based solely on images. Hence, researchers have recently devoted substantial effort to developing methods for computer-aided diagnosis (CAD) [15].

Convolutional neural networks (CNNs), belonging to deep learning technology, are automatic feature extraction learning algorithms that generally extract image features via convolution and pooling layers. Subsequently, the images are classified based on the extracted features [16]. Through a multi-level non-linear transformation, they can transform the initial "bottom" feature representation into a "high-level" feature representation, and then use a "simple model" to complete complex classification and regression tasks. By contrast, a traditional machine learning algorithm has difficulty in processing the original data, and usually needs to artificially extract features from the original data. This requires a system designer to have a fairly professional knowledge of the original data. Transfer learning, refers to pre-training a neural network model on a very large data set (e.g., ImageNet), and then re-training a small number of trained layers for application to a new task on a limited data set, is one of the most successful deep learning algorithms [17,18]. With transfer learning, this architecture makes it possible to process images in the form of pixels as input, and to provide the desired classification as output [19]. As compared with computer vision, the number of pieces of medical data is not too large, so CNNs with transfer learning are now being increasingly used in medical image analysis.

Research regarding CNNs for CAD has expanded to include CXRs [20], computed tomography (CT), and high-resolution CT [21]. Besides widespread application in computer vision and image classification tasks [22], CNNs are increasingly being utilized in radiology and medical image analysis for diagnosis. Some examples include diagnosis of pediatric pneumonia through chest X-ray images [23], detection of pulmonary tuberculosis [24] and pneumothorax [25] in chest radiography, mediastinal lymph nodes in CT, lung nodules in CT and brain segmentation [26–28]. What’s more, the technology is also widely used in the field of cancer pathology diagnosis[29], such as predict microsatellite instability directly from histology in gastrointestinal cancer [30], classification and mutation prediction from non-small cell lung cancer histopathology images [31]. In a recent study, deep learning technology was adopted to evaluated cancer therapy, for example, to assess tumour-infiltrating CD8 Cells and response to anti-PD-1 or anti-PD-L1 immunotherapy [32].

However, up to date, no previous studies have used CNNs to predict PH based on CXR images. In this study, we used CNN models to identify possible abnormalities on CXRs suggesting PH, with the purpose to screen potential PH patients while minimizing the need for additional radiographic examination.

## Methods and materials

### Study population

This is a multi-center retrospective study comprised of three hospitals, including the Third Affiliated Hospital of Sun Yat-sen University, the Third Affiliated Hospital of Sun Yat-sen University. Yuedong Hospital, and Dongguan Tangxia Hospital. Private records for all participants were anonymized prior to analysis. The institutional review boards of the hospitals that participated in this retrospective study approved the study.

**Fig 1:**
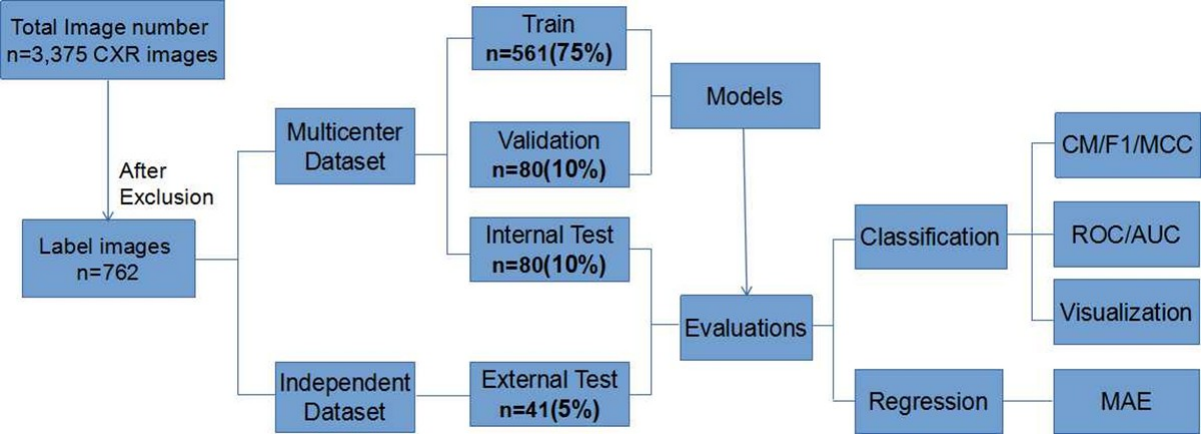
Workflow diagram illustrating the overall experimental design. It described the flow of CXR images through labeling process followed by training transfer learning models using multicenter data and evaluating the models with internal multicenter testing data and independent external testing data. The labeled images were chosen from the initial total dataset according to diagnostic criteria and sufficient quality. CM, confusion matrix; MCC, Matthews correlation coefficient; ROC, receiver operating characteristic; AUC, the area under the curve; MAE, mean absolute error.

Frontal images from the above three institutes were identified by searching image databases for CXRs of the patients who also received a doppler TTE test within 3 days, whereas lateral radiographs and oblique views were excluded. Candidate CXRs of adult patients were obtained from 1 January 2013 to 30 April 2019. In that regard, 3375 frontal chest radiographic images obtained during that period with a Doppler TTE test were identified. From those images, 755 patients with PH were selected according to the results of the TTE, i.e., with PASP ≥ 40 mmHg. After exclusion of images with pleural effusion, pneumothorax, pulmonary atelectasis, pericardial effusion, massive pulmonary consolidation, or obstruction of the right ventricular outflow tract or pulmonary artery, 405 patients with PH were included in the study. Meanwhile, 357 control patients were also selected, according to normal results of the TTE and radiology reports. These 762 subjects composed the sample of this study (41 from Yuedong Hospital, 102 from Dongguan Tangxia Hospital, and 619 from the Third Affiliated Hospital of Sun Yat-sen University). The 721 patients from the latter two hospitals were mixed as a new dataset, and then were randomly split into a training set (641) and an internal test set (80), with a ratio of 89%:11%. Of the 41 cases from Yuedong Hospital used for the external test, 20 were healthy, and the remaining 21 were patients with PH. We firstly performed a 8-fold cross-validation on the 641 images used for final-training (561 for pre-training, 80 for validation), and then decided to tune learning rate to 0.0008 according to the best score on validation data. Finally, we used all the pre-training and validation data (561+80 = 641) to train our models and evaluated them on internal and external data according to the area under the receiver operating characteristic curve (AUC/ROC). The overall workflow of this study is illustrated in Fig 1.

### Deep learning and transfer learning methods

**Fig 2:**
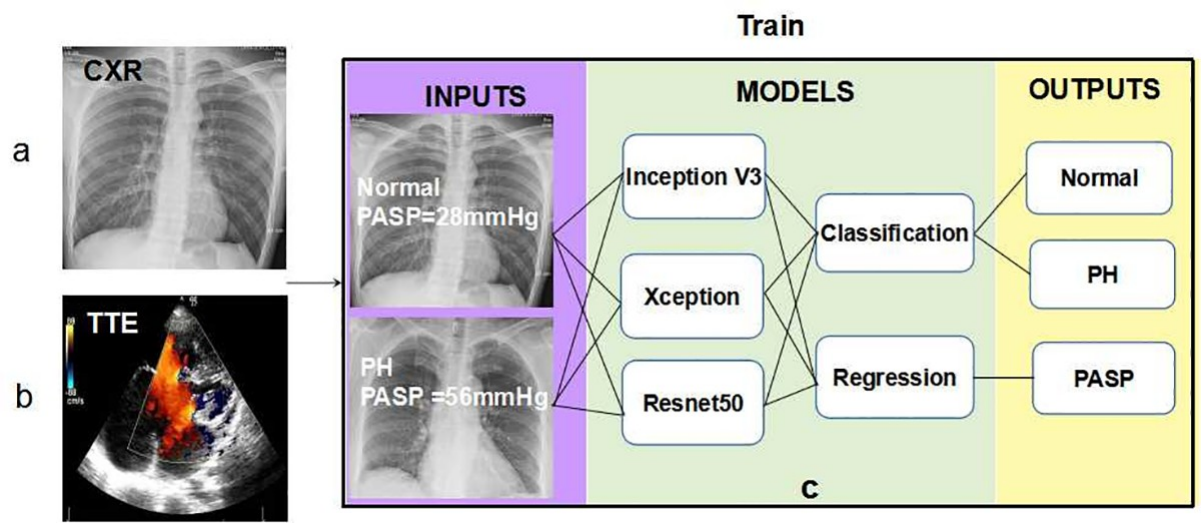
Schematic of labeling and training process. A Patient with or without PH receiving CXR screening (a). The same patient receiving TTE to identify PH by measuring PASP value with cutoff of 40 mmHg (b). Using retrospective CXR and corresponding PASP tags to train transfer learning models that can be deployed to diagnose PH and predict PASP according to the CXR of new patients (c). CXR, chest X-ray; TTE, transthoracic echocardiography; PH, pulmonary hypertension; PASP, pulmonary artery systolic pressure.

Training deep learning model needs a large amount of data. Compared to natural images such as Imagenet, our sample sized was relatively small, so we adopted transfer learning to obtain an effectively trained deep learning model and then fine-tuned the parameters based on images of CXR. In this study, we compared three commonly used architectures (Resnet50, Xception, and Inception V3) for applying transfer learning algorithms, to assess their performance on the classification and regression of CXR images. Each model first loaded weights pre-trained on the ImageNet dataset, and then removed their top layer. Next, for the task of diagnosing PH with a classification algorithm, a full-connection layer containing two neurons was added, and the softmax activation function was used. For the task of predicting exact PASP value with a regression algorithm, a full-connection layer containing only one neuron was added. No activation function was used at this time, to ensure that the model had a broader output value. Finally, the parameters of all layers of the new model architecture were fine-tuned according to the input images and corresponding labels. The process of deep learning is shown in Fig 2.

To compare the performance accuracy achieved by deep learing models with that of manual classification, we invited a board-eligible chest radiologist with 15 years’ experience to classify the images as PH or not in the test dataset (including internal and external test). The expert was blind and had no access to the deep-learning predictions. The manual classification results were recorded and compared with the that obtained by deep learning models.

### Implementation details

In this research, we adopted the Keras (version 2.2) framework using a Tensorflow (version 1.8) backend within the Python (version 3.6) programming language, including libraries such as numpy, matplotlib, and Scikit-learn, to train and evaluate our models. The computing power was provided by one Tesla V100 GPU with 32 GB memory on an Nvidia DGX1 server, which had eight Tesla V100 GPUs, 512 GB DDR4 memory, and 7 TB SSD. Since the focus of our research was on the lung and heart regions, we firstly cut the original CRX images to remove the edges outside the thoracic cavity and patients’ information, then uniformly adjusted the size of the images to 512 x 512 pixels, converted these into floating-point tensors, and re-scaled the pixels (between 0 and 255) to the (0, 1) interval, and saved them in the JPG format. In order to prevent over fitting to some extent, we randomly rotated images within degrees (0, 20) to augment the training data only.

### Statistical analysis

To assess the performances of various architectures and radiologist for classification tasks on a sample dataset, we chose AUC/ROC as our primary evaluation measurement. A commonly used confusion matrix was used to visually evaluate the performances of the deep learning algorithms. Each row of the matrix represented an instance in the real label, and each column represented an instance in the prediction label, as shown in S1 Fig. In addition to AUC/ROC, we performed sensitivity analyses to better evaluate the top model’s potential clinical utility. The sensitivity, specificity, positive predictive value (PPV) and negative predictive value (NPV) were calculated based on the confusion matrix, while F1-score was obtained by the index above (F1 = 2*precision*recall/(precision+recall), and Matthews correlation coefficient (MCC) value was also calculated. The ROC curve was depicted by plotting the true positive rate (TPR, sensitivity) against the false positive rate (FPR, 1 -specificity) at various threshold settings. The accuracy was measured by AUC/ROC.

To quantify and evaluate the performances of various models of regression tasks on the same dataset, the mean absolute error (MAE) was computed. The MAE is a measure of the difference between two continuous variables (e.g., PASP). SPSS16.0 was also used for statistical analyses. Students’ t tests were used to compare continuous variables, and a Pearson chi-square test was used to compare categorical variables. *P* values <0.05 were considered statistically significant.

$$\mathrm{MAE}(y,\hat{y})=\frac{1}{n_{\mathrm{samples}}}\sum_{i=0}^{n_{\mathrm{samples}}–1}{|y}_{i}–{\hat{y}}_{i}|$$

## Results

### Participants

Females accounted for 52.6% (401) of all the patients included in this study, and males for 47.4% (361), with an average age of 59.9 years. While the gender between patients with or without PH showed no significant difference, the mean age in patients with PH was (67.5 ± 14.8) years, as compared to (49.4 ± 16.5) years in those without PH, with *P* <0.05.

### Convolutional neural networks model performance

**Fig 3:**
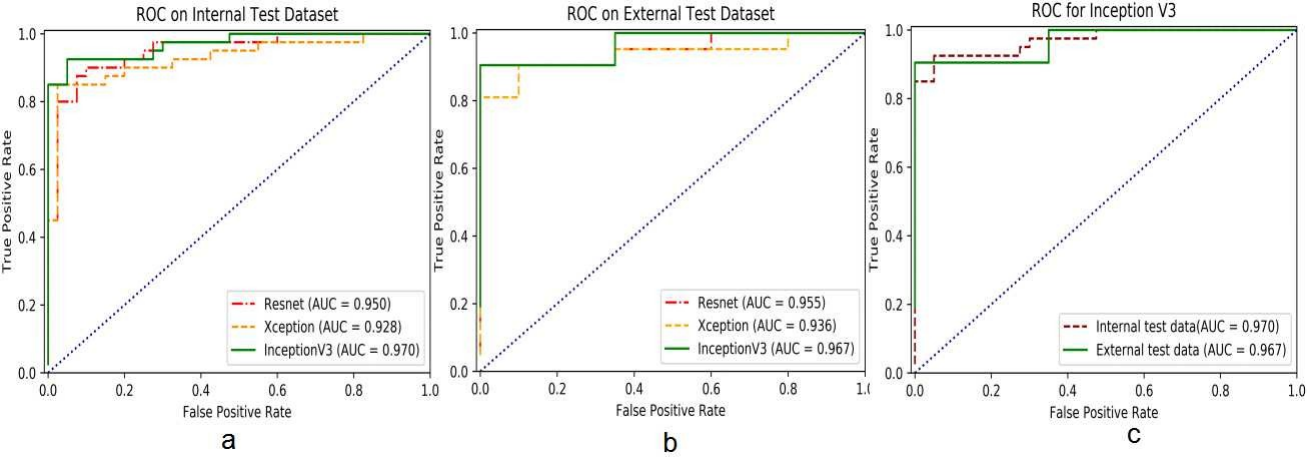
The AUC/ROCs for detection of PH from normal. Comparision of AUC/ROCs in internal test. The InceptionV3 model had an AUC (0.970) greater than the other two models (a). Comparision of AUC/ROCs in external test. The InceptionV3 model provided the best AUC (0.967) compared to the other ones (b). The AUC performed by InceptionV3 model in external test was slightly lower than that in internal test (0.967 VS 0.970 respetively) (c). ROC, receiver operating characteristic; AUC, area under the ROC curve; PH, pulmonary hypertension.

Three CNN models (Resnet50, Xception, and InceptionV3) were used in the present analysis to distinguish PH from normal. The AUC/ROCs of the above three models to distinguish PH from normal were 0.950, 0.928, and 0.970 in internal test, compared to 0.955, 0.936, and 0.967 in external test, respectively. The best AUCs in both test sets were provided by the InceptionV3 model (shown in Fig 3). The AUC value for InceptionV3 model was 0.967 in the external test, just slightly lower than that in the internal test (0.970), indicating a good generalizability of this CNN model.

In internal test, the InceptionV3 model yielded a greatest sensitivity (0.93) and PPV (0.93) respectively compared to the Resnet50, and Xception models, while the best specificity (0.97) and NPV (0.97) were performed by Xception model. For the external test set, the confusion matrix showed that the InceptionV3 model also provided a highest sensitivity of 0.9 and best NPV (0.90), when the best specificity (1.0) was achieved by Resnet50 model, which also had a best PPV of 1.0 compared to other two models. Both the F1-score and MCC values were higher in internal test than that in external test for Resnet50 model. Nevertheless, the situation for the other two models (Xception and InceptionV3) were opposite, which showed higher F1-score and MCC in external test compared to that in internal test (as shown in Table 1).

**Table 1:** Comparison of performances of deep learning and manual in internal and external test.

	Internal test				External test			
	Resnet50	Xception	InceptionV3	Manual	Resnet50	Xception	InceptionV3	Manual
Sensitivity	0.90	0.85	0.93	0.62	0.86	0.86	0.90	0.62
specificity	0.90	0.97	0.95	0.97	1.0	0.90	0.90	0.95
PPV	0.90	0.97	0.95	0.96	1.0	0.90	0.90	0.93
NPV	0.90	0.87	0.93	0.72	0.87	0.86	0.90	0.70
F1-score	0.90	0.91	0.94	0.79	0.92	0.88	0.90	0.77
AUC	0.950	0.928	0.970	0.80	0.955	0.936	0.967	0.785
MCC	0.80	0.832	0.876	0.64	0.858	0.757	0.805	0.60

Abbreviations: PPV, positive predictive value; NPV, negative predictive value, AUC, area under the ROC curve, MCC, Matthews correlation coefficient.

The confusion matrix (S2 Fig) indicated that manual classification of PH based on CXR had similar specificity and PPV value as deep learning models, but much lower sensitivity, NPV, F1-score and MCC. In addition, both the AUCs for manual prediction of PH in internal test (0.80) and exteranl test (0.785) were far smaller than that performed by deep learning models (as shown in Table 1).

**Fig 4:**
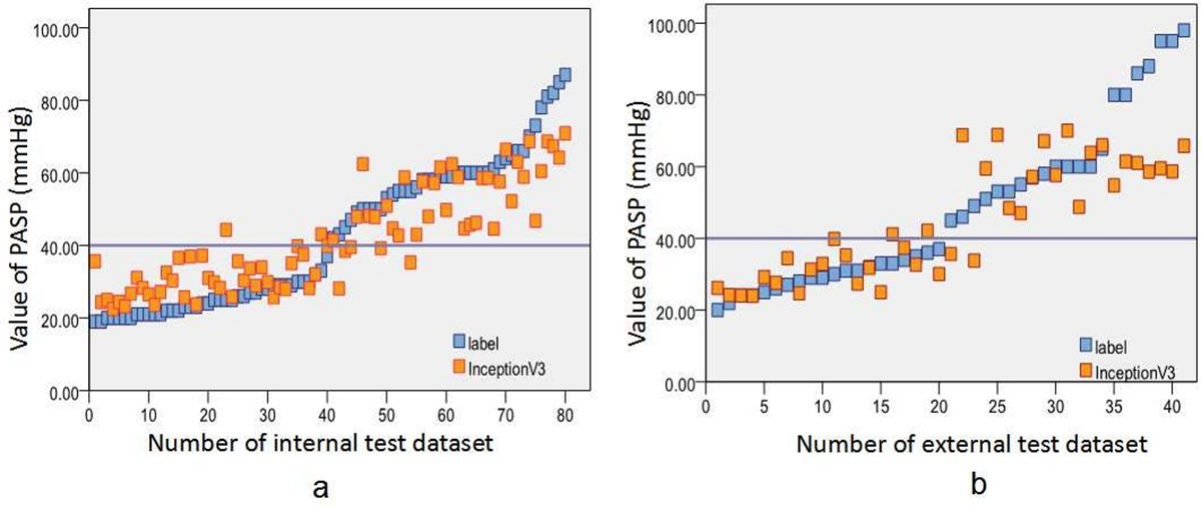
Prediction of exact PASP value by best model (InceptionV3) based on chest radiographs. Prediction of exact PASP value by InceptionV3 model in internal test, with a MAE of 7.45 (a). Prediction of exact PASP value by InceptionV3 model in external test, and the MAE was 9.95 (b). PASP, pulmonary artery systolic pressure; MAE, mean absolute error.

We further used the CNNs to predict exact PASP value. The results of the regression prediction showed that the InceptionV3 model exhibited a smaller MAE in both internal (7.45) and external tests (9.95) when compared to the MAEs produced by the other two models (illustrated in S3 and S4 Figs), indicating that this CNN model performed best in predicting PASP value through detection from CXR. Moreover, the whole three CNN models performed better overall in the internal test than in the external test when predicting PASP value based on CXR, as the MAEs obtained by the Resnet50, Xception, and InceptionV3 models were greater in external test than those in the internal test (an average of 10.13 versus 7.85, respectively). The increase of MAE from the internal test to external test suggested that the intensity of prediction declined when using the external data. The results are summarized in Table 2, and the predictions of exact PASP values by the InceptionV3 model are shown in Fig 4.

**Table 2:** Comparison of MAEs for regression prediction of PASP in internal and external test.

	InceptionV3	Resnet50	Xception	Average
MAEs of internal test set	7.45	8.79	8.18	7.85
MAEs of external test set	9.95	10.45	10.01	10.13

Abbreviations: MAE, Mean absolute error; PASP, pulmonary artery systolic pressure.

## Discussion

The present study employed a transfer learning algorithm to detect abnormalities suggesting PH from chest radiographs. Our models retained high performance in accuracy, sensitivity, specificity in both internal and external tests even with a relatively limited training dataset, highlighting that the models had considerable potential to provide an initial screening of PH based on CXR images.

**Fig 5:**
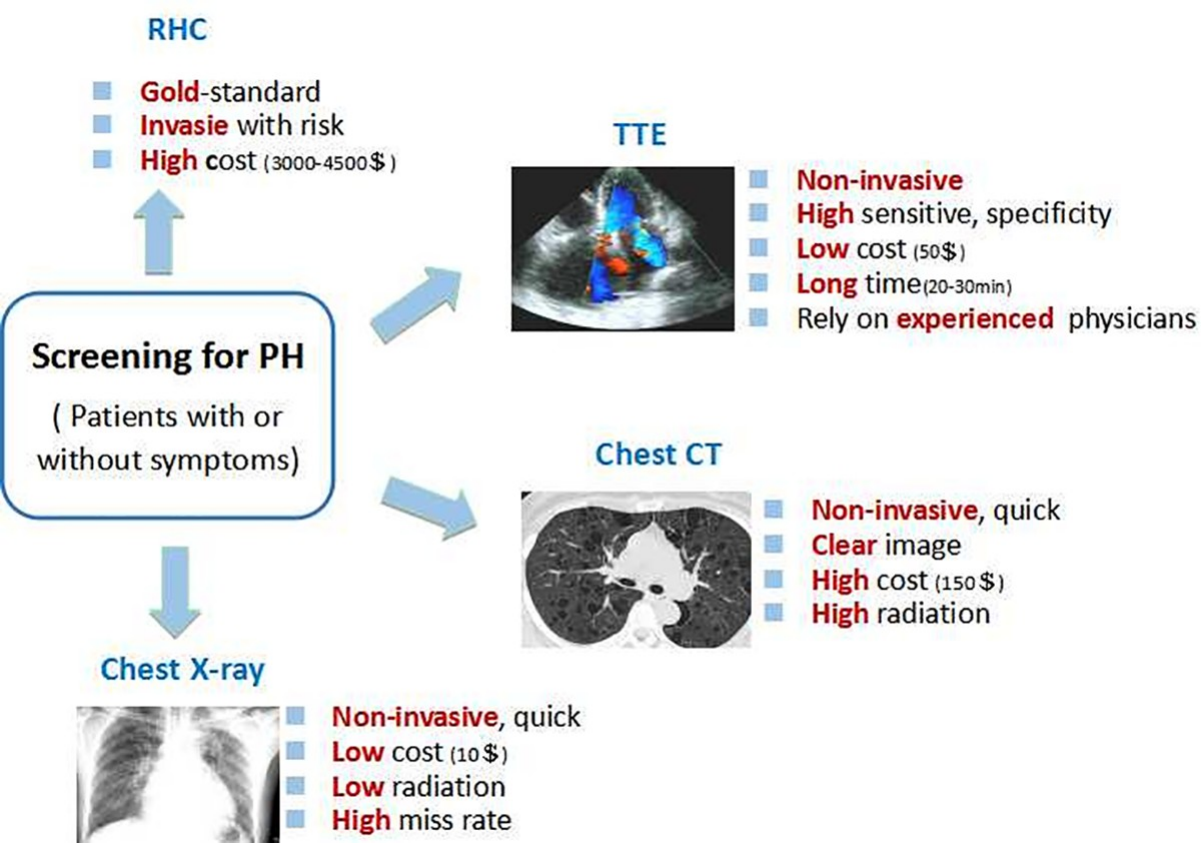
Comparision of the advantage and defect of different ways to screen spicious PH patients. PH, pulmonary hypertension; RHC, right heart catheterization; TTE, transthoracic echocardiography; CT, computed tomography.

As we know, sever PH may lead to heart failure and death if left untreated [3]. Hence, it is of great clinical significance to find a simple, quick, and noninvasive way to screen the disease at an early stage. RHC it is not considered as the first-line examination for clinically screening PH for invasiveness and high risks in addition to high cost. Doppler TTE is recommended by the current guidelines to be performed in the initial evaluation whenever PH is suspected. Even so, this method is difficult to perform in some poor areas in developing countries, where it’s in a sever shortage of ultrasound equipment and experienced ultrasounl physicians due to poor economy, as the examination of TTE is relative expensive while requiring more time and needs large quantities of ultrasound physicians compared to chest X-ray. Chest CT scan, another important method for diagnosis and evaluation of PH, is also infeasible for screening PH, owing to the high radiation exposure and economic cost (As shown in Fig 5). For comprehensive consideration, CXR is the most common and useful way for screening PH eventhough rapid radiologic interpretation of images is not always available as well as relative high miss rate by radiologist, both can be improved with the help of CNNs. Recent advances in machine deep learning have enabled radiologists to extract more information from CXR images and significantly improved the identification and classification of diseases based on CXRs while minimizing the need for additional radiographic examination [33].

In this study, three models were adopted. For the best-performing inceptionV3 model, the AUC value was nearly the same both in internal and external test (0.970 versus 0.967, respectively) when predicting whether a patient was PH positive or negative (PASP ≥ 40 mmHg or < 40 mmHg). This demonstrated that the model had a high degree of generalization. Our work expands and improves upon some prior studies [25,34], which used a dataset from a monocenter for model training, as the data in the present study was built based on multi-centers, and the dataset used for training was also from more than single center. This was one of the determinant factor to keep good generalizability by promising the CNN models to perform well in transfering from the training step. As a result of that, the AUCs performed by these models did not decline in further tests based on independent datasets in the external test. Nevertheless, when we used the same models to predict the exact PASP values based on the CXR images, the average MAEs in the external test were significantly greater than those in the internal test (10.13 versus 7.85, respectively). The situation for the best-performing inceptionV3 model remained the same, with a MAE of 9.95 in external test, and 7.45 in internal test. This result meant that the generalization became weak when it came to prediction of exact PASP through CXR images. This was in concordance with our research aim, which was meant to be a screening and triaging tool for potential PH patients, rather than a substitute evaluation and diagnosis by a human radiologist through TTE or RHC. Thus, the automated model is not intended to be relied upon for diagnosis of PH, or to judge the therapy effect of PH patients who have been given medicines for lower PAP.

We also compare the performance accuracy achieved by deep learing models with that of manual classification. Our result showed that manual classification of PH based on CXR had much lower values of AUC, sensitivity, NPV, F1-score and MCC compared to that performed by deep learning models both in internal and external test. It further indicated that screeing PH by radiologist based on CXR had rather high miss rate. Deep learning technology can improve this situation and increase the sensitivity of classifying PH based on CXRs.

**Fig 6:**
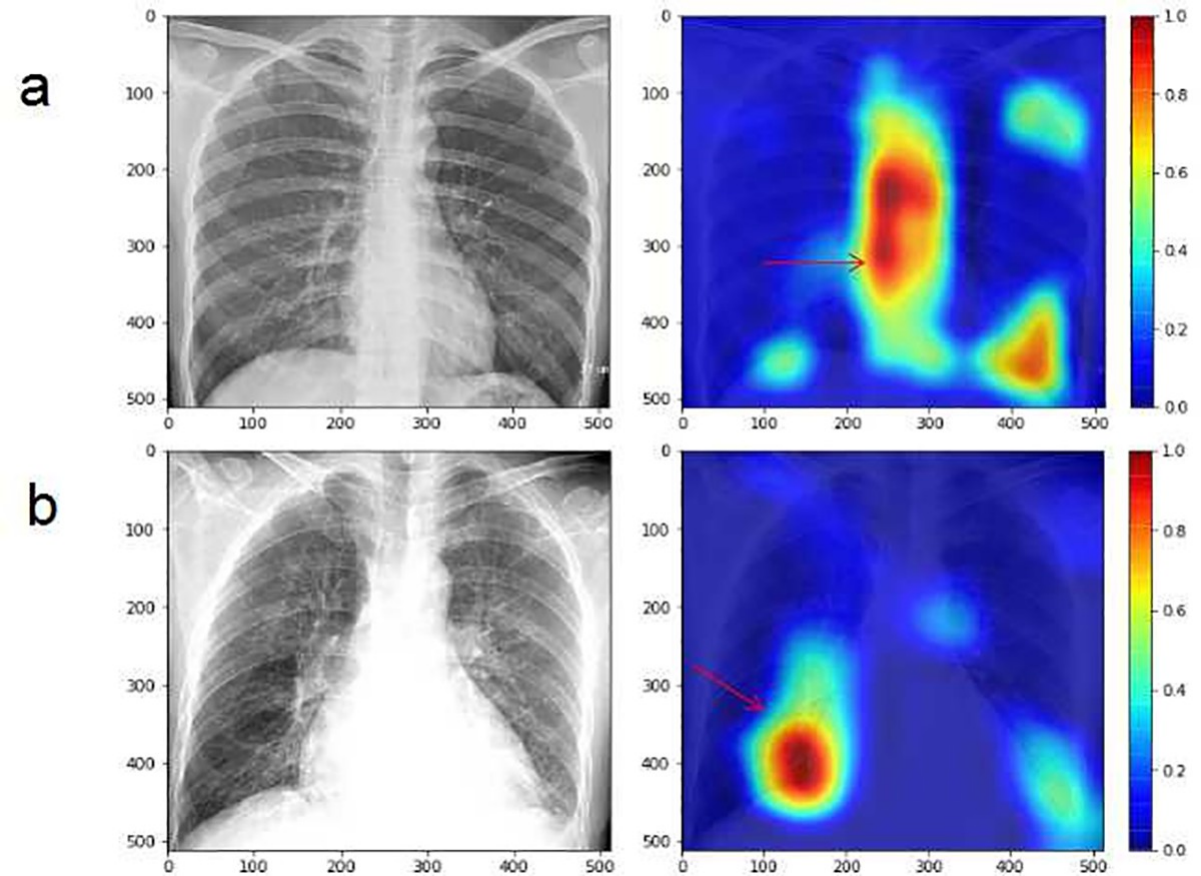
Visualization of CNN models using Grad-CAM to classify PH from normal based on radiograph images. The highlighted areas indicatied by red arrows are discriminative features for identification of PH. The Grad-CAM of patient without PH (a). The Grad-CAM of patient with PH (with PASP of 56mmHg) (b). CNN, Convolutional Neural Network; PH, pulmonary hypertension; Grad-CAM, Gradient Class Activation Map; PASP, pulmonary artery systolic pressure.

Gradient-weighted Class Activation Mappings (Grad-CAMs) were used to visualize areas that the model predicted to be most indicative in the radiographs of each observation in the current study. Usually evaluation criterias can help us get to know whether the performance of models is in accordance with requirements on classification and regression tasks, yet they cannot explain the basis for making such a judgment, which is quite important for clinical diagnosis. In contrast, CNN models can better explain the results and display them visually, as they uses Grad-CAM to translate the output class into final convolutional layer to produce a low-resolution map for a particular category (e.g., PH and Normal), and highlight the discriminative image pixels and regions used by the models to identify that category [35]. With the help of Grad-CAM, we can judge whether the classification basis of the model is consistent with the medical diagnostic criteria. Some examples of the Grad-CAMs are illustrated in Fig 6. The models localized the areas of the hilum or central pulmonary arteries and the right heart chamber, which were discriminative features for classifying PH from radiographs. However, the figure also showed highlighting of areas other than those, indicating the existence of overfitting, even though this did not have a significant impact on the detection results.

Overfitting is one of the most common problems in the use of deep learning in medicine, because a massive number of parameters are involved in the process of learning, making it difficult to identify the specific variables determining predictions. Thus, overfitting occurs [36]. This means that the trained model fits the training data well, but does not generalize well to test data. This occurs more commonly when the training size is not large enough. To avoid this, we followed standard practices in training deep learning models to ensure internal generalization between the training data and held-out test data. Moreover, the subjects included in the present study were divided into three groups (training, internal test set, and external test set). The AUC/ROCs of the classifiers were based on the external test dataset, which had not been learned by the trained networks, indicating that the algorithm was generalizable, and could provide accurate results with new cases.

As reported in previous studies, building a balanced dataset of categories is an important issue for consideration when using CNNs, especially when the goal is to detect relative rare pathologies like severe PH, as there are often not enough examples available to form a robust training set to match other categories [37,38]. In this study, the results of regression prediction analysis showed that the prediction error became larger in patients with PASP ≥ 80 mmHg when using CNNs to evaluate PASP values through CXR images, and the output value was not sufficiently consistent with the PASP measured by Doppler TTE. One possible reason was that the number of patients with PASP over 80 mmHg was relatively small, which might have resulted in training insufficiency, and then affected the prediction accuracy. Moreover, the network is likely to be biased toward the classes that it had seen more when the class imbalance existed. In the present study, the number of patients with severe PH or PASP over 80 mmHg in the training set was smaller than the number of normal samples and/or those with mild or moderate PH, which might have led to a bias of the CNNs toward mild to moderate (or even normal) categories. However, determining independent optimal thresholds for a positive result by analysis of ROC analysis could help to offset this outcome.

There were several limitations in the present study. The first was that radiologic diagnoses are usually made in the context of a patient’s history and clinical presentations more than images only. Positive findings on a chest radiograph are necessary but not sufficient for a final diagnosis of PH. In the present study, the CNN models for image classification were trained using images only. Although the gender distribution showed no difference, the mean age was significantly different between participants with PH-positive and PH-negative. Such differences may have certain impacts on results of deep learning and general statistical analysis, suggesting that training the CNN models based on a combination of multiple demographic variables and CXR images may significantly improve test performance. Second, the relatively small sample size might have limited our ability to detect external performance degradation in some cases. Nevertheless, many key comparisons achieved statistical significance with even this smaller external dataset.

## Conclusions

This was a retrospective study using deep learning algorithms to detect abnormalities suggesting PH in chest radiographs. Clinical integration of this system could allow for a transformation of simple and quick screening of PH, especially in poor areas of developing countries. Further studies are necessary to determine the feasibility of these outcomes in a prospective clinical setting.

## Supporting information

S1 Fig Confusion matrix of different CNN models in internal and external test. Confusion matrix of different CNN models for internal test set (a); Confusion matrix of different CNN models for external test set (b). (0: subjects without PH; 1: subjects with PH). CNN, Convolutional Neural Network; PH, pulmonary hypertension.
(PDF)

S2 Fig Confusion matrix of manual classification of PH in test dataset. Confusion matrix of manual classification of PH in internal test set (a); Confusion matrix of manual classification of PH in in external test set (b). PH, pulmonary hypertension.
(JPG)

S3 Fig Prediction of exact PASP value by different models in internal test set. InceptionV3 model, with a MAE of 7.45 (a); Xception model, with a MAE of 8.18 (b); Xception model, with a MAE of 8.79 (c); Prediction of exact PASP value averge, with a MAE of 7.85 (d). PASP, pulmonary artery systolic pressure; MAE, mean absolute error.
(JPG)

S4 Fig Prediction of exact PASP value by different models in external test set. InceptionV3 model, with a MAE of 9.95 (a); Xception model, with a MAE of 10.01 (b); Xception model, with a MAE of 10.45 (c); Prediction of exact PASP value averge, with a MAE of 10.13 (d). PASP, pulmonary artery systolic pressure; MAE, mean absolute error.
(JPG)
