# Supplementary figures and images for: A promising approach for screening pulmonary hypertension based on frontal chest radiographs using deep learning: A retrospective study
Source: PLoS One. 2020 Jul 24;15(7):e0236378. doi: 10.1371/journal.pone.0236378 (PMC7380616; doi:10.1371/journal.pone.0236378)

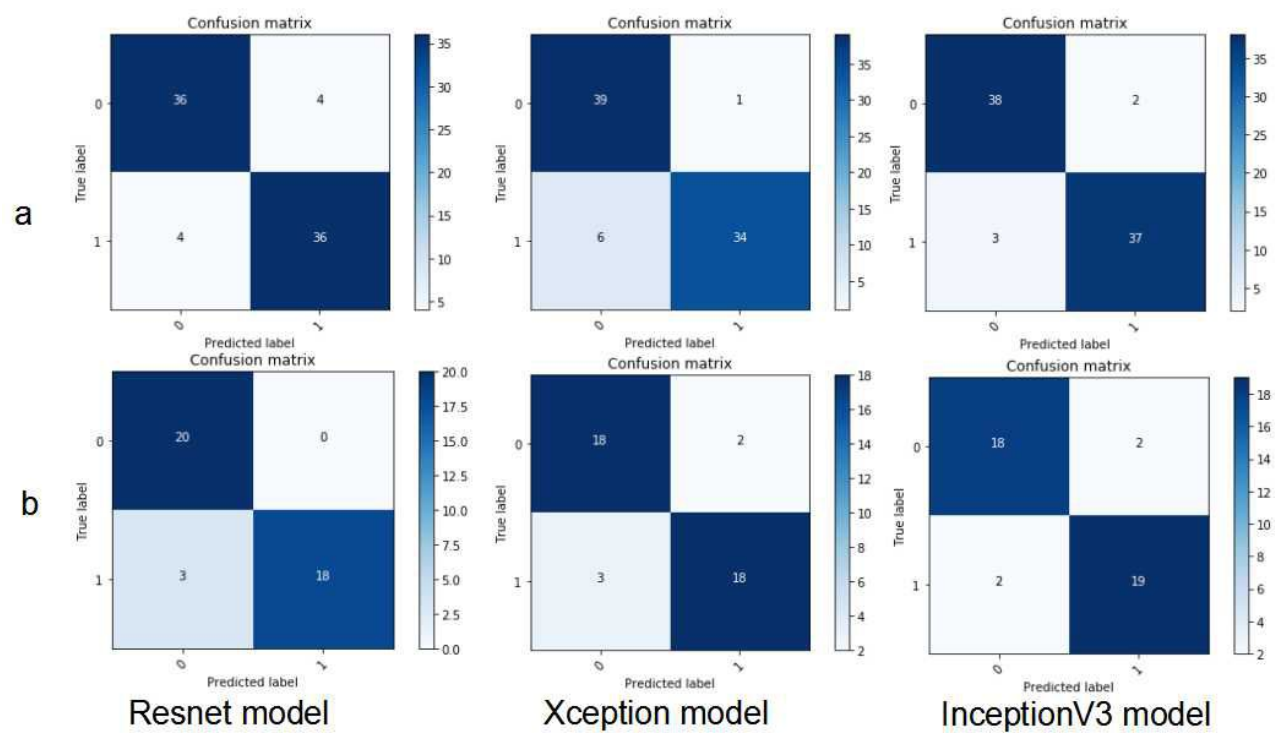

**S1 Fig. Confusion matrix of different CNN models in internal and external test.**

Supplement: S1 Fig — Confusion matrix of different CNN models for internal test set (a); Confusion matrix of different CNN models for external test set (b). (0: subjects without PH; 1: subjects with PH). CNN, Convolutional Neural Network; PH, pulmonary hypertension. (PDF) [file pone.0236378.s001.pdf]

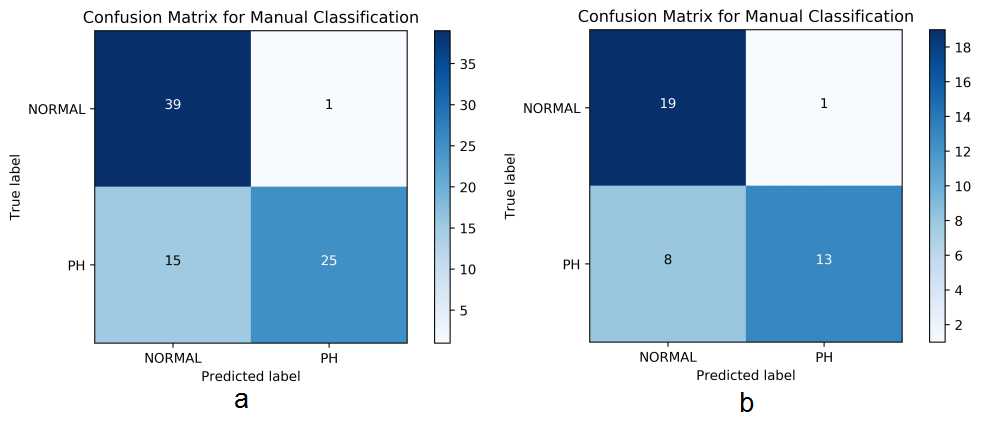

Supplement: S2 Fig — Confusion matrix of manual classification of PH in internal test set (a); Confusion matrix of manual classification of PH in in external test set (b). PH, pulmonary hypertension. (JPG) [file pone.0236378.s002.jpg]

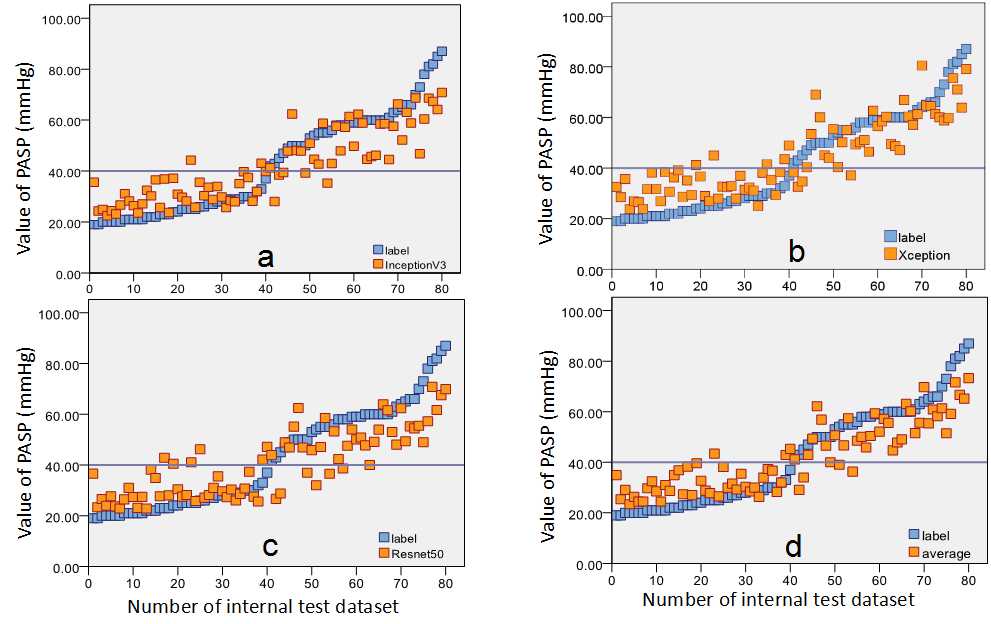

Supplement: S3 Fig — InceptionV3 model, with a MAE of 7.45 (a); Xception model, with a MAE of 8.18 (b); Xception model, with a MAE of 8.79 (c); Prediction of exact PASP value averge, with a MAE of 7.85 (d). PASP, pulmonary artery systolic pressure; MAE, mean absolute error. (JPG) [file pone.0236378.s003.jpg]

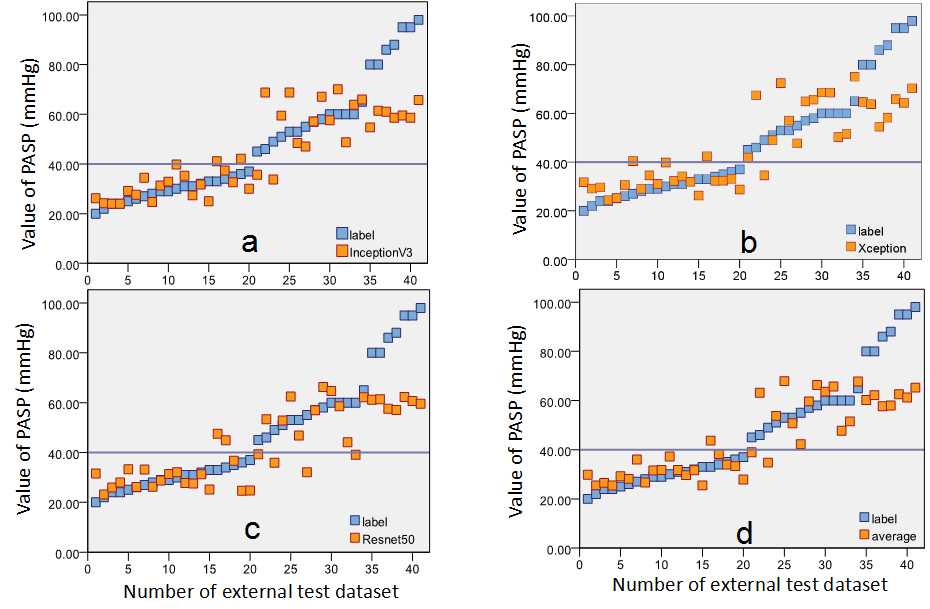

Supplement: S4 Fig — InceptionV3 model, with a MAE of 9.95 (a); Xception model, with a MAE of 10.01 (b); Xception model, with a MAE of 10.45 (c); Prediction of exact PASP value averge, with a MAE of 10.13 (d). PASP, pulmonary artery systolic pressure; MAE, mean absolute error. (JPG) [file pone.0236378.s004.jpg]
